# Recovery of physical activity levels in adolescents after lower limb fractures: a longitudinal, accelerometry-based activity monitor study

**DOI:** 10.1186/1471-2474-13-131

**Published:** 2012-07-25

**Authors:** Dimitri Ceroni, Xavier Martin, Léopold Lamah, Cécile Delhumeau, Nathalie Farpour-Lambert, Geraldo De Coulon, Victor Dubois Ferrière

**Affiliations:** 1 Pediatric Orthopedic Unit, Department of Child and Adolescent, University of Geneva Children's Hospital and University of Geneva Faculty of Medicine, 6 Rue Willy Donzé, 1211, Geneva 14, Switzerland; 2 Clinical Epidemiology Service, Department of Child and Adolescent, University of Geneva Children's Hospital and University of Geneva Faculty of Medicine, 6 Rue Willy Donzé, 1211, Geneva 14, Switzerland; 3 Exercise Medicine, Pediatric Cardiology Unit, Department of Child and Adolescent, University of Geneva Children's Hospital and University of Geneva Faculty of Medicine, 6 Rue Willy Donzé, 1211, Geneva 14, Switzerland

**Keywords:** Adolescent, Motor activity, Bone disease, Fracture, Body weight changes

## Abstract

**Background:**

In adolescents, loss of bone mineral mass usually occurs during phases of reduced physical activity (PA), such as when an injured extremity spends several weeks in a cast. We recorded the PA of adolescents with lower limb fractures during the cast immobilization, at 6 and at 18 months after the fracture, and we compared these values with those of healthy controls.

**Methods:**

Fifty adolescents with a first episode of limb fracture and a control group of 50 healthy cases were recruited for the study through an advertisement placed at the University Children’s Hospital of Geneva, Switzerland. PA was assessed during cast immobilization and at 6- and 18-month follow-up by accelerometer measurement (Actigraph® 7164, MTI, Fort Walton Beach, FL, USA). Patients and their healthy peers were matched for gender and age. Time spent in PA at each level of intensity was determined for each participant and expressed in minutes and as a percentage of total valid time.

**Results:**

From the 50 initial teenagers with fractures, 44 sustained functional evaluations at 6 months follow-up, whereas only 38 patients were studied at 18 months. The total PA count (total number of counts/min) was lower in patients with lower limb fractures (-62.4%) compared with healthy controls (*p*<0.0001) during cast immobilization. Similarly, time spent in moderate-to-vigorous PA was lower by 76.6% (*p*<0.0001), and vigorous PA was reduced by 84.4% (*p*<0.0001) in patients with cast immobilization for lower limb injuries compared to healthy controls values. At 6 and 18 months after the fracture, the mean PA level of injured adolescents was comparable to those of healthy teenagers (-2.3%, and -1.8%, respectively).

Importantly, we observed that time spent in vigorous PA, which reflects high-intensity forces beneficial to skeletal health, returned to similar values between both groups from the six month follow-up in adolescents who sustained a fracture. However, a definitive reduction in time spent in moderate PA was observed among patients with a lower limb fracture at 18 months, when comparing with healthy controls values (*p* = 0.0174).

**Conclusions:**

As cast immobilization and reduced PA are known to induce bone mineral loss, this study provides important information to quantify the decrease of skeletal loading in adolescents with limb fractures. The results of this study demonstrate that the amount of skeletal loading returns to normal values in adolescents with lower limb fractures after bone healing and is probably linked to an overall better pattern of functional recovery among this age group. When comparing both populations of adolescents, a definitive decrease in time spent in moderate-to-vigorous PA was observed among patients with a lower limb fracture at 18 months and may suggest a modification of lifestyle. The high rate of missing data (26.5%) due to above all non compliance with monitor wearing among teenagers complicates the data analysis, and requires a more cautious interpretation of the results. Future studies using accelerometer to monitor PA in adolescents should therefore include strategies for improving the rate of adherence and minimizing the ratio of missing data.

## Background

Physical activity is essential for bone remodeling. The skeleton needs continuous physical stimulation to maintain healthy bones, otherwise bone loss ensues [1]. In childhood, exercise exerts a positive effect on bone growth, especially if the activity has been initiated before puberty or in the early pubertal period [2]. Bone mineral mass is higher in children who are physically active than in those who are mildly active [3], and higher in children who participate in activities that generate high impact forces than in those who engage in activities that impart lower impact forces [4-6]. Studies have shown that high-intensity forces, especially when imposed during early childhood, produce greater gains in bone mass than low- to moderate-intensity forces [7-11]. Based on this evidence, it is now recommended that physical activity for children include activities generating relatively high ground-reaction forces, such as jumping, skipping, running, and possibly strengthening exercises [12].

It is also recognized that lack of normal stress to the bone can result to osteopenia [13-15], the physiological response of the bone to disuse. Osteopenia has been defined in a pathogenic manner as an uncoupling in the bone remodeling in which resorption exceeds formation resulting in a net loss of bone [16]. In children and adolescents, loss of bone mineral mass usually occurs during phases of reduced PA, such as when an injured extremity spends several weeks in a cast[17]. In a previous study, we demonstrated that there was a significant reduction of the PA level among subjects with lower limb fractures during cast immobilization [18].

Using total activity measurement, subjects with lower limb fractures were considerably less active (-62.4%) than healthy fracture-free controls [18], and above all, the time spent in vigorous PA, which reflects high-intensity forces beneficial to skeletal health, demonstrated an 84.4% decrease in adolescents with lower limb fractures [18]. However, once the injury has healed and normal stresses are restored to the extremity, the bone stock recovers to a large extent, although the period of recovery is several times longer than the period of bone loss, and the degree of recovery shows wide individual variation [19].

If a full recovery from post-traumatic osteopenia seems not possible in adults [20-22], it is expected in children and in adolescents [2,23,24]. In this regard, the pattern of better bone recovery in young patients is probably linked to a better pattern of functional recovery among this age group. Clinical experience shows that children and adolescents demonstrate a faster functional rehabilitation than adults in daily life. The major components explaining this phenomenon include probably better flexibility, muscle strength, muscular balance, agility, and functional coordination. All these parameters improve body controls among adolescents and help to achieve a full return to their pre-injury level by accelerating the mechanical loading of the skeleton [25].

To the best of our knowledge, measurement of PA levels by accelerometry has never been explored among adolescents after bone healing of lower limb fractures to corroborate this assumption. The purpose of this study was thus to elucidate whether PA levels normalize after bone healing in this population.

## Methods

### Participants

We conducted a longitudinal matched case–control study between January 2005 and December 2008 including 50 adolescents with a lower limb fracture and 50 healthy paired controls (no past history of fracture) aged 10 to 16 years. Injured adolescents and healthy controls were recruited for the study through an advertisement placed at the Children’s Hospital of the University of Geneva Hospitals, Geneva, Switzerland. Injured adolescents were selected if they were admitted to hospital as inpatients for orthopedic reduction or minimal invasive surgery (closed reduction and stabilization by percutaneous wires or screws) of their fracture under general anesthesia. Patients who required an open reduction of their fracture with hardware placement were not eligible for inclusion. Participants were required to wear a long cast and be non-weight-bearing during the initial healing phase, and agree to be followed up at the orthopedic clinic during the whole duration of the study (from fracture time until 18 months follow-up). Exclusion criteria for both injured adolescents and healthy controls were: prior history of bone fractures; chronic disease; congenital or acquired bone disease; any condition limiting physical activity; and hospitalization for more than 2 weeks in the previous 12 months. All participants and their parents provided written consent and the protocol was approved by the institutional ethics committee (protocol # 04-057, ped 04-002).

### Anthropometric measurements

Standing height was measured in bare or stocking feet using a precision mechanical stadiometer (Holtain Ltd, Dyfed, UK), and body weight by a mechanical Seca® calibrated beam scale (Seca, Reinach, Switzerland). Body mass index (BMI) was calculated as weight in kilograms (kg) divided by height in meters squared (m^2^).

### Physical activity measurement

PA was measured during the cast immobilization, and thereafter 6- and 18 months after the fracture. Objective measures of PA were obtained using an uniaxial accelerometer (Actigraph® 7164, MTI, Fort Walton Beach, FL, USA). The monitor was set on a 1 min cycle; at the end of each run, the sum was stored in the memory and the numerical integrator reset. Monitors were attached above the iliac crest of the right hip with an elastic belt and adjustable buckle, and were oriented vertically in the same direction. Accelerometers were programmed to start recording at 8 am on the first day of measurement and participants were asked to wear them continuously for 10 days. Recordings of physical activity started on Monday, Tuesday, or Wednesday to ensure measurement of two weekend days. Data collection was conducted during all seasons. As we did not have criteria defining the normal physical activity in adolescents, data emanating of matched healthy controls (matching for age and gender) were regarded as the normative values.

### PA data interpretation

Data reduction was based on criteria applied in previous publications [26-29]. Only periods between 8 am and 9 pm were analysed. Zero activity periods of 20 min or longer were interpreted as being due to unworn accelerometers and were removed from the activity totals [30]. Participants who did not manage to record more than 600 min d^-1^ of activity [27,29,31-33] for at least 5 days were excluded from further analysis [34]. Data were expressed as total activity counts per registered time (counts.min^-1^) to generate an average range of PA. We used the cut-offs of intensity levels described by Ekelund where sedentary behaviour was defined as less than 500 counts.min^-1^, light PA from 500 to 1999 counts.min^-1^, moderate PA from 2000 to 2999 count.min^-1^, and vigorous PA as > 3000 counts.min^-1^[28]. Time spent at each PA intensity category was also determined for all participants as a percentage of total valid time.

### Statistical methods

Pairs were matched for age (+/- 6 months) and for gender (male/female). Data are expressed as mean ± (SD). A paired Student’s *t* test with an alpha threshold of 5% was used to analyze the variability of matching characteristics (age and gender) for cases and healthy controls. A Shapiro Wilk test with an alpha threshold of 5% was used to test the normality of PA variables. As these variables did not have a normal distribution, a paired Wilcoxon test with an alpha threshold of 5% was used to assess differences of PA levels between cases and healthy controls. We decided not to use imputation of the missing values, if the rate of missing data was superior to 10% of the values, since we considered that excluding rows prevents any error from being introduced due to the missing values. Data analyses were performed using STATA 9.2 (StataCorp LP, Texas, USA).

## Results

### During cast immobilization

Fourteen children with lower limb fractures were excluded either for failing to reach at least 5 days of measurement or for instrument malfunction. Thirty for patients with lower limb fractures were able to be matched with 34 healthy controls. Age, physical characteristics, and PA levels of those with fractures and healthy controls are presented in Table 1. There was no statistical difference between groups for age, height, weight, BMI, or daily duration of PA monitoring. As expected, cases with lower limb fractures showed notable reductions in PA levels and spent more time in sedentary activities. The total PA count (number of counts/min) was significantly lower in those with lower limb fractures (-62.4%) compared with healthy controls. When considering time spent in moderate-to-vigorous PA, we observed lower levels in lower limb (-76.6%) fracture groups compared to matched healthy controls. Finally, there was a significant reduction of vigorous PA in the injured group compared to healthy controls (-84.4%). All these data are similar to those allready published in an anterior paper about the subject [18].

**Table 1:** Characteristics and Physical Activity Measures of Adolescents with Lower Limb Fractures during Cast Immobilization vs Healthy Controls

	**Injured adolescents**	**Healthy controls**	***p*value**
	**(n = 34)**	**(n = 34)**	
Age (yr)	13.6 ± 1.6	13.3 ± 1.6	0.2312
Height (cm)	161.9 ± 11.1	162 ± 12.9	0.9501
Weight (kg)	52.1 ± 11.8	51.8 ± 11.8	0.8128
BMI (kg/m^2^)	19.7 ± 2.5	19.5 ± 2.5	0.2614
Number of valid monitored days (days)	9.1 +/- 1.8	8.2 +/-2	0.0276*
Daily duration of physical activity monitoring (min)	746.7 ± 22.9	749.3 ± 33.3	0.8241
Total activity (counts/min^.^day)	200.9 ± 92.3	534.5 ± 207.3	<0.0001*
Time spent in sedentary activity (min/day)	664.4 +/- 40.9	564.3 +/- 64.4	<0.0001*
(% of total daily wearing time)	89	75.3	
Time spent in light activity (min/day)	68.4 +/- 26.4	125.7 +/- 40.1	<0.0001*
(% of total daily wearing time)	9.2	16.8	
Time spent in moderate (min/day)	8.9 +/- 7.2	27.5 +/- 11.4	<0.0001*
(% of total daily wearing time)	1.2	3.7	
Time spent in vigorous activity (min/day)	5 +/- 6.8	31.8 +/- 18	<0.0001*
(% of total daily wearing time)	0.6	4.2	
Time spent in moderate to vigorous activity (min/day)	13.9 +/- 13.4	59.4 +/- 28.4	<0.0001*

The results are expressed as mean ± SD. *: statistically significant difference (please always place at foot of table).

### At 6 months follow-up

Forty-four injured adolescents and their matched peer controls underwent accelerometer measurement 6 months after the fracture. Twelve adolescents were excluded either for failing to reach at least 5 days of measurement or instrument malfunction. Finally, 32 adolescents with lower limb fractures and their matched peer healthy controls were included in the study. Age, physical characteristics, and PA levels of adolescents with fractures and healthy controls are shown in Table 2. There was no statistical difference between groups for age, height, weight, BMI, daily duration of PA monitoring, or mean number of valid monitored days. Adolescents with lower limb fractures demonstrated same PA level as healthy controls. However, patients with lower limb fractures demonstrated less time spent in moderate PA (-23.3%, *p* = 0.0174). We did not note any significant difference between injured adolescents and healthy controls for time spent in vigorous PA. Similarly, there was no significant difference between both groups for time spent in activities of moderate-to-vigorous intensity, and the time spent in these activities (59.1 min per day) reached the international guidelines’ recommendations for school-age children (at least 60 min of moderate-to-vigorous intensity activity daily [35].

**Table 2:** Characteristics and physical activity measures of teenagers with a 6-month past history of lower limb fractures vs healthy controls

	**Injured teenagers**	**Healthy controls**	p value*
	**(n = 32)**	**(n = 32)**	
Age (y)	13.6 ± 1.5	13.5 ± 1.6	0.4547
Height (cm)	161.0 ± 10.9	162.6 ± 12.6	0.3883
Weight (kg)	52.4 ± 9.3	53.5 ± 10.8	0.3698
BMI (kg.m^-2^)	20.1 ± 1.9	20.1 ± 2.6	0.9556
Number of valid monitored days	7.9 +/- 2.3	7.7 +/- 2.1	0.7905
Daily duration of the physical activity monitoring (min)	745.4 ± 32.4	746.4 ± 35.8	0.9989
Total activity (counts.min^-1^.d^-1^)	556 ± 266.9	569.2 ± 219.7	0.7366
Sedentary activity (min/day) (%)	563.4 +/- 57.7	554.4 +/- 69.2	0.7548
	75.6	74.3	
Light activity (min/day) (%)	122.8 +/- 38.1	127.1 +/- 42.9	0.6654
	16.5	17	
Moderate (min/day) (%)	23.2 +/- 10	30.2 +/- 13.8	0.0174*
	3.1	4.1	
Vigorous activity (min/day) (%)	35.9 +/- 19.5	34.7 +/- 18.5	0.7731
	4.8	4.6	
Time spent in moderate to vigorous activity (min.day^-1^)	59.1 ± 37.2	64.9 ± 30.9	0.3366

The results are expressed as mean ± SD. *: statistically significant difference [please always place at foot of table].

### At 18 month follow-up

Thirty-eight injured adolescents and their matched peer controls were reviewed at 18 months’ follow-up, and pairing was possible with healthy controls in 20 cases due to data reduction and pair-matching procedures and, above all, to a higher rate of patients refusing to wear the accelerometer. Age, physical characteristics, and PA levels of cases with fractures and healthy controls are shown in Table 3. There was no statistical difference between groups for age, daily duration of PA monitoring, or the mean number of valid monitored days, but those who had sustained a lower limb fracture had a significantly higher BMI (*p* = 0.0012). Adolescents with lower limb fractures demonstrated same PA level as healthy controls. As previously, those with lower limb fractures still demonstrated a residual significant reduction of time spent in moderate PA (-30.5%; *p* = 0.0086) compared to the values of healthy teenagers. In addition, time spent in activities of moderate-to-vigorous intensity was significantly less than 60 min (51 min), thus no longer reaching the international guidelines’ recommandations for school-age children [35]. However, we observed that there was no significant difference between injured adolescents and healthy controls for time spent in vigorous PA.

**Table 3:** Characteristics and physical activity measures of teenagers with an 18-month past history of lower limb fractures vs healthy controls

	**Injured teenagers**	**Healthy controls**	p value*
	**(n = 20)**	**(n = 20)**	
Age (y)	14.3 ± 1.7	14 ± 1.8	0.0764
Height (cm)	167.6 ± 12.7	162.8 ± 13.5	0.1239
Weight (kg)	60.6 ± 14.9	55.3 ± 13.4	0.0156*
BMI (kg.m^-2^)	21.2 ± 2.8	20.6 ± 3.1	0.0012*
Number of valid monitored days	7.2 +/- 2.8	8.5 +/- 2	0.1541
Daily duration of the physical activity monitoring (min)	736.7 ± 25.1	744.4 ± 27.8	0.3812
Total activity (counts.min^-1^.d^-1^)	531.1 ± 256.5	541 ± 216.5	0.4925
Sedentary activity (min/day) (%)	577.9 +/- 44.5	563.5 +/- 62.1	0.9058
	78.5	75.7	
Light activity (min/day) (%)	107.8 +/- 27.3	119 +/- 34.7	0.4776
	14.6	16	
Moderate (min/day) (%)	20.1 +/- 7.5	28.9 +/- 10.8	0.0086*
	2.7	3.9	
Vigorous activity (min/day) (%)	30.9 +/- 24.7	33.1 +/- 18.9	0.4631
	4.2	4.5	
Time spent in moderate to vigorous activity (min.day^-1^)	51 ± 28.9	62 ± 28.2	0.1024

The results are expressed as mean ± SD. *: statistically significant difference [please always place at foot of table].

## Discussion

Most skeletal fractures involving the lower limb are treated non-operatively with cast immobilization and represent therefore a frequent cause of reduction of the PA level in healthy children and adolescents. The most predictable consequences of cast immobilization and subsequent weight-bearing restriction are loss of bone mineral tissue, substantial muscle atrophy, and corresponding functional limitations. A full recovery from post-traumatic osteopenia is expected in children and in adolescents (personnal data submitted for publication), and this fast mineral accretion is intuitively attributed to an easier and faster weight bearing observed among these age groups. Nevertheless, there are currently no data to prove that physical activity levels really normalize among children and teenagers who sustain a lower limb fracture. To the best of our knowledge, this is thus the first study to report PA measures in a representative sample of an adolescent population after bone healing of lower limb fractures.

In a previous study, we demonstrated that there was a significant reduction of the PA level among subjects with lower limb fractures during cast immobilization [18]. Using total activity measurement, subjects with lower limb fractures were considerably less active (-62.4%) than healthy fracture-free controls [18]. This decrease in PA was even more marked for time spent in activities of moderate-to-vigorous intensity, since the average time spent at this level of PA was 13.9 min for adolescents with lower limb fractures [18]. Our study investigated also the time spent in vigorous PA, which reflects high-intensity forces beneficial to skeletal health, and demonstrated an 84.4% decrease in adolescents with lower limb fractures [18]. The significant reduction in high-intensity forces applied to the skeleton brought thus a valid explanation for disuse osteopenia noted in the same patients [17].

These results further our comprehension of recovery from post-traumatic osteopenia. As mentioned previously, restoration of bone mineral mass in childhood may occur upon reaching pubertal maturity[36,37] and, above all, normal activity [38]. In fact, the patterns of the bone recovery in young patients appear probably to be linked to a better pattern of functional recovery in this patient population [25]. Our results seem confirm this hypothesis, since there was a fast normalization of the time spent in vigorous PA after cast removal, which reflects high-intensity forces beneficial for bone mineral accrual. Therefore, it can be concluded that restoring the mechanical loading caused by PA can stimulate bone formation and restore the bone mineral mass very rapidly after cast removal (personnal data submitted for publication).

The present study revealed that adolescents with lower limb fractures were less active and demonstrated a significant decrease of time spent in moderate-to-vigorous intensity activity at 18 month follow-up (51 min). International guidelines recommending that school-age youth should participate daily in at least 60 min of moderate-to-vigorous intensity PA to prevent weight gain and to increase bone mineral mass [39]. The reduction noted in MVPA (-15% at 18 months follow-up) among patients after a lower limb fracture seems to be definitive and suggests thus a modification of lifestyle. The reduction of PA in adolescents with fractures results in a decrease of energy expenditure and our clinical experience has shown that this decrease during the cast immobilization period could be the starting point for children and adolescents to become overweight. In this series, we noted that adolescents with lower limb fractures developed a significant increase of BMI at the end of the follow-up compared to healthy controls. Unfortunately, as the study’s drop-out rate among patients at 18-month follow-up was very important (> 50%), these findings are less robust than those at 6 months follow-up and need to be interpreted with caution.

Other limitations should be considered when interpreting the findings of the present study. Firstly, there are activities during which accelerometers have to be removed (swimming) or do not accurately measure the intensity of exercise (cycling). These “unmonitored” activities may result in underestimations of PA. Nevertheless, Trost et al. stated that the children’s self-reported periods of “unmonitored” activity added to the registered accelerometer data led to no significant changes in calculated activity levels [40]. Secondly, to obtain 10 days of recordings, activity counts were averaged using a 1-minute epoch in order to ensure that the accelerometer’s memory capacity was not exceeded. This method of averaging underestimates the teenager’s VPA because such activity is rarely sustained for longer than 1 minute [33]. Previous studies have demonstrated that VPA may be substantially undervalued [24]. However, the underestimation of VPA is unlikely to be important in this study, as we can hypothesize that it will be the same for the different groups. Thirdly, we had to omit 31 cases (26.5%) of the recordings from the analysis, because data originated from obviously defective accelerometers (10 cases), or failure to reach the minimum number of days required or the minimum number of minutes per day to validate recordings (21 cases), whereas 15 teenagers refused to wear the accelerometer (cf Table 4). Our figure of 26.5% exclusion (31 cases on 117 recordings) is largely higher to data published by Pate et al. [41], who reported a figure of 6.3% exclusion over a 7-day recording period, but very close to the 25% exclusion rate over a 3-day period reported by Riddoch.[33] The greatest limitation when interpreting the findings of the present study is due both, to the the high percentage of missing data (26.5%) and to the high drop-out rate à 18 months among study’s participants (60%). Finally, we did not record the pubertal status in this study as we did not consider it was a consistent contribution factor to the prediction of PA. In fact, many European studies could not find any associations of pubertal status with PA for both genders. For this reason, the pubertal status was not recorded and this omission was not considered as a limitation of this study [42-44].

**Table 4:** Characteristics of missing data and lost of FU

	**Baseline data (fracture time)**	**6 months FU**	**18 months FU**
Participants nbr	50	44	38
Lost of FU	0	6	12
Drop-out rate among study’s participants	0%	12%	24%
Missing data	16	12	18
Rate of missing data	32%	27.3%	47.4%

Accelerometer use in physical activity research has become increasingly popular, but it is challenging among teenagers and it is prone to problems with missing data, which complicate the data reduction and analysis process. Many studies have focused on the optimal approach for obtaining high adherence and low levels of missin data. Applying adherence-promoting strategies, such as approaching the research participant as a partner, making a personal connection with teachers, parents, and coaches, using different reminder means (reminder stickers, cell phone features), and above all providing an adequate incentive payement may have a positive impact on wearing the monitor and data qualities [41].

## Conclusion

There are general challenges to the use of accelerometers in teenagers, including noncompliance with monitor wearing or even monitor loss; therefore, accelerometer use in PA assessment is prone to problems with missing data, that may complicate data reduction and results analysis. Therefore, future studies using accelerometry to monitor PA should include adherence enhancing strategies specially when dealing with teenagers. Nevertheless, PA measured by accelerometer remains a valid tool to quantify the decrease of PA level of adolescents with lower limb fractures during immobilization, and to assess the recovery of PA level. As recovery of PA levels is known to induce bone mineral accretion, this study provides important information to estimate the restoration of skeletal loading in adolescents with lower limb fracture. However, further research is needed to establish the relationship between the PA level and bone mineral acquisition during cast immobilization and after bone healing. Finally, this study brings a hypothetical explanation of the weight gain observed in many children during the follow-up of lower limb fractures.

## Competing interests

The authors declare that they have no competing interests.

## Authors’ contributions

Dimitri Ceroni: participated in the design of the study, conceived and coordinated the study, collected and treated the patients, performed the CSA analysis, and drafted the manuscript. Xavier Martin: conceived the study, participated in its coordination, collected the data, and performed the statistical analysis. Léopold Lamah : collected and treated the patients and drafted the manuscript. Cécile Delhumeau: performed the statistical analysis and drafted the manuscript. Nathalie Farpour-Lambert: participated in the design and conception of the study, and drafted the manuscript. Geraldo De Coulon: collected and treated the patients and drafted the manuscript. Victor Dubois-Ferrière: participated in the design, collected and treated the patients, and drafted the manuscript. All authors read and approved the final version of the manuscript.

## Pre-publication history

The pre-publication history for this paper can be accessed here:

http://www.biomedcentral.com/1471-2474/13/131/prepub
